# Analysis of Occlusal Vertical Dimension and Mandibular Basal Bone Height in a Nigerian Population

**DOI:** 10.1155/2014/584508

**Published:** 2014-10-02

**Authors:** Babatunde O. Akinbami, Prince E. Nsirim

**Affiliations:** ^1^Department of Oral and Maxillofacial Surgery, University of Port Harcourt, Rivers State, Port Harcourt 500004, Nigeria; ^2^Department of Human Anatomy, University of Port Harcourt, Rivers State, Port Harcourt 500004, Nigeria

## Abstract

*Background*. The actual basal bone height of the reconstructed mandible is relevant to achieve normal occlusal vertical dimension for the prosthesis fabricated. The purpose of the study was to determine the mean and baseline values of the occlusal vertical dimension and height of the mandibular basal bone in a Nigerian population.* Method*. Each participant was asked to bring the upper and lower teeth into contact, while the distance between the nasal sill and dimple on the lower lip was measured (OVD). The skin at lower border of the mandible was marked and the distance between this point and the landmark on the lower lip was measured, MBH.* Result*. 200 subjects were evaluated. Age range was 16–30 years, mean ± (SD), 21.6 ± (3.1) years. Males had mean ± (SD) of 42.10 ± (5.34) mm for OVD and females 39.72 ± (5.25) mm; acceptable baseline range of OVD for any population will be 34–48 mm (3.4–4.8) cm. All the males had a mean ± (SD), 30.54 ± (6.13) mm for MBH, and all the females 29.63 ± (5.23) mm. Acceptable baseline range of MBH for any population will be 24–37 mm (2.4–3.7) cm.* Conclusion*. To reconstruct the mandible and still maintain the OVD, heights of bone grafts must not be less than 2 cm or greater than 4 cm.

## 1. Introduction

The mandible is a horseshoe shaped bone and the body of the mandible on either side has the basal bone component and the mandibular arch embedding teeth. The head of the mandibular condyle articulates with the glenoid fossa to form the temporomandibular joint. Morphological changes of the mandible are thought to be influenced by the occlusal status and age [1]. The adult human mandible is a bone which exhibits a large degree of anatomical variability. This variation occurs not only between subjects or as a result of aging, but also between the right and left sides in an individual. During facial growth, the maxilla and mandible translate downward and forward. Although the forward displacement of the maxilla is less than that of the mandible, the interarch relationship of the teeth in the sagittal view during growth remains essentially unchanged [2]. Interdigitation is thought to provide compensatory (tooth movement) mechanism for maintaining the pattern of occlusion during growth; the maxillary teeth move anteriorly relative to the maxillary basal bone while the mandibular teeth move posteriorly relative to the basal bone of the mandible (Marshall et al., 2011). After growth has ceased, the single most important factor governing the gross morphological shape of the bone is related to the presence or absence of the teeth. After tooth extraction, there follows a phase of remodeling which may result in an extensive loss in the height of the jaws, particularly the mandible [3].

The occlusal vertical dimension relates the maxillary alveolar arch/teeth with the mandibular alveolar arch/teeth and it is the distance between these components with the upper and lower teeth/jaws interdigitating [4]. The basal bone of the mandible can be entirely reconstructed following resection of tumors or avulsion due to trauma. In such instances, it is important to know the actual basal bone height to be reconstructed, which will also be adequate to achieve normal occlusal vertical dimension for the prosthesis fabricated. When the basal bone height is increased, the occlusal vertical dimension (OVD) will be reduced; there will be functional and esthetic problems, difficult lip contact, speech problems, and also temporomandibular joint pain dysfunction. Also, if the reconstructed mandibular basal bone height is reduced, the OVD will increase, and overclosure will also occur with difficulty in bringing the upper and lower teeth into contact, dribbling of saliva, cheek biting, and myofascial/temporomandibular joint pain dysfunction [5]. The aim of the study was therefore to determine the mean and baseline values of the occlusal vertical dimension and height of the mandibular basal bone in a young adult Nigerian population.

## 2. Methods

This was a prospective study carried out in the Departments of Human Anatomy and Oral/Maxillofacial Surgery, University of Port Harcourt, Rivers State, between January and March, 2014. The study was carried out on 200 subjects from different tribes in Nigeria. This included volunteer subjects within the age limit of 16–30 years with complete anterior and posterior natural dentition. Excluded were those with multiple tooth (incisors) extraction and those with congenital malformation affecting the face and jaws. Informed consent was obtained from all the subjects. Subjects were seated in a comfortable upright position. The nasal sill at the point of attachment of the columella to upper lip was marked with a pen; this point corresponds to the level of maxillary alveolar bone. On the lower lip, the point just below the depression between the free upper part and the attached lower part of the lip which corresponds to the level of the mandibular alveolar bone was also marked. Each participant was asked to bring the upper and lower teeth into contact, while the distance between these two points was measured. This represented the occlusal vertical dimension (OVD).

**Figure 1:**
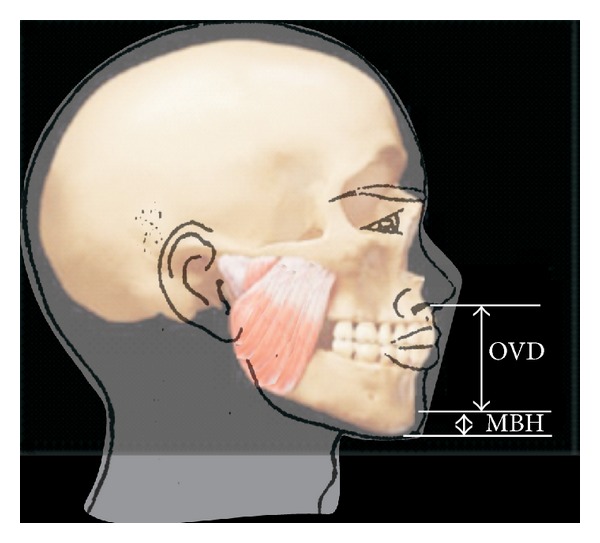
Showing the landmarks for measurements of OVD and MBH.

The skin overlying the symphysis mentum (lower border) of the mandible was also marked and the distance between this point and the landmark on the lower lip was also measured. This represented the mandibular basal bone height, MBH (Figure 1). A caliper was used to measure these distances on the face to the nearest 0.1 mm, and the measurements were recorded. The measurement was taken at least twice to ensure reliability. The data obtained was analyzed using SPSS version 16, SPSS, IL, Chicago. Data was expressed as simple frequencies and proportions. Means and standard deviations were determined, comparison of means of OVD and MBH between the genders for each age group was done with paired sample* t*-test, and *P* value less than 0.05 was considered significant. Also means in both genders in the whole sample population was determined and the baseline range of values of OVD and MBH for any given population was set, CI 95%.

## 3. Results 

A total of 200 subjects were evaluated during the study period. Age range was 16–30 years, means ± (SD), 21.6 ± (3.1) years. The highest number of subjects, 134 (67%), recorded OVD between 40 mm and 49 mm while 113 (56.5%) subjects recorded MBH between 30 mm and 39 mm. The lowest number of subjects, 3 (1.5%), recorded OVD between 20 mm and 29 mm while 8 (4.0%) subjects recorded MBH between 40 mm and 49 mm (Table 1).

Both OVD and MBH mean values were higher in males than females in all age groups. The mean value of MBH was highest in the 21–25-year age group in both genders while the 26–30-year age group in males recorded the highest OVD. The significance level for mean values of OVD for males and females in the 16–20 years group was compared, it was 0.000. However, there was no statistical significant difference between the mean values of OVD in both genders in the 21–25- and 26–30-year age groups, *P* > 0.05; 0.655; also for MBH, there was no statistical significant difference, *P* > 0.05; 0.847, 0.656, and 0.139 (Table 2).

In our sample population, minimum value for OVD was 29.00 mm and maximum value was 53.00 mm. All the males had a mean ± (SD) of 42.10 ± (5.34) mm for OVD and all females had a mean of 39.72 ± (5.25) mm; acceptable baseline range of OVD for any population will be 34–48 mm (3.4–4.8) cm.

In our sample population, minimum value for MBH was 20.00 mm and maximum value was 47.00 mm. All the males had a mean ± (SD) of 30.54 ± (6.13) mm for MBH, and all the females had a mean ± (SD) of 29.63 ± (5.23) mm. Acceptable baseline range of MBH for any population will be 24–37 mm (2.4–3.7) cm (Table 3).  Table 4 shows comparisons of OVD values in this study with previous studies.

## 4. Discussion

Occlusal vertical dimension is not exactly the same as the lower facial height because denture bases/flanges do not reach the inferior border of the mandible. Different reference landmarks accounted for the differences in values documented in this present study in comparison with the other tabulated previous studies. Also, many studies have documented that loss of posterior teeth reduces alveolar height but they have only determined the relationship of the height of the mandibular and maxillary alveolar processes and how changes in either affect the occlusal vertical dimension, Abduo and Lyons [4]; Marshall et al., 2011; Ural et al., [6]. In our study, we have assessed differences in both the MBH and the OVD in various age groups, which no study has addressed. The importance of this study is best reflected clinically when the grossly atrophic or resected mandible is to be reconstructed to achieve the normal height of the mandibular basal bone that will support prosthetic device taken into consideration.

From our findings, the mean description of subjects showed that occlusal vertical dimension and mandibular bone height increase with increasing age up to age 25, after which there was a slight decline; this may be attributable to reduced growth after immediate postpubertal ages, and increasing masticatory forces in older age groups. However, there was still a direct proportionality existing between age and the measured variables for the adolescents, and this was consistent with findings of Marshall et al., 2011.

The gender mean comparison showed that both occlusal vertical dimension and mandibular bone height are higher in male than female subjects; therefore supporting Ural et al., [6] that decrease in the height of the edentulous mandible was more pronounced in women than in men. Reconstruction of the atrophic, avulsed, or tumor affected mandible must take consideration of the rest vertical dimension (OVD + freeway space) and occlusal vertical dimensions, and from this study, bone graft heights of 2.0 cm to 3.0 cm will be sufficient to maintain the occlusal vertical dimensions in many patients.

Ali [7] carried out a study on comparison between skeletal and facial measurements of vertical dimension in edentulous patients. Evaluation of data showed that the 3 mm skeletal distance and the 5 mm skeletal distance would be represented as 1.81 mm and 3.55 mm mean value as measured on reference marks on the face, respectively. This was in agreement with the study done by Huang et al. [1], who found that the 3 mm mandibular opening represents 1.90 mm mean value as measured on the skin. In addition, this result agrees with the study made by Chiaki et al., who made measurements with dividers between reference marks above and below the oral commissure on subjects while their natural teeth were in occlusion and when they were at known constant intraocclusal separation maintained by acrylic splints. They concluded that measurements between marks on the face were less than the mandibular opening involved. In addition, they reported that what was being measured was not an alteration in skeletal relationships but concomitant independent alteration in the relative position of soft tissue.

However the present study disagrees with Robert [8], who suggested that a freeway space of 3 mm in the premolar region would be represented as 4-5 mm if measured on the skin of the face. The present study clearly indicates that the reference marks on face move when interridge distance increases, but the hard and soft tissue do not move to the same extent. This finding is coincident with that of Hansen [9], who found considerable differences between skin markers and bone references on cephalometric radiographs. Moreover, this study is supported by the results of Balshi and Wolfinger [10], who found a greater interocclusal distance recorded by the tooth attached reference than with a chin attached reference point. Despite conflicting evidence in the literature regarding the measuring of the vertical dimension in edentulous patients, the use of facial reference points is still a popular method in clinical practice, and both the caliper and the Willis gauge techniques are used in research studies.

In restoring lost vertical dimension of occlusion using dental implants Balshi and Wolfinger [10] gave a clinical report that the decision to sacrifice the remaining mandibular anterior teeth and place osseointegrated dental implants may be considered radical treatment. However, if some of the mandibular anterior teeth had been preserved, fixed partial denture restorations would have been necessary to restore proper form, reduce the vertical overlap, and increase the occlusal vertical dimension. Many methods can be used to determine OVD based on vertical dimension in rest position of the mandible or phonetics, but none is better than the other, so a combination of the methods is commonly used; however, implant retained restorations preserve the reestablished occlusal vertical dimension over longer period than conventional dentures.

At times there may be need to alter occlusal vertical dimension and such alterations may be a compatible modality of management and this may improve esthetics, facial height, and effective bite force management in the masticatory system [11]. These alterations will be individual specific and it will have effect on the rest vertical dimension, TMJ loading, tooth loading, and neuromuscular stability [12].

In many cases it is possible to increase the vertical dimension of occlusion if 2 foundational principles are maintained. First, the starting point for reconstruction of the vertical dimension of occlusion must be with the mandibular condyles in centric relation. Second, reconstruction must be within the range of neuromuscular adaptation for each individual patient. The difficulty is determining both of these parameters on an individual patient basis, accurately recording the centric reference point and transferring this information to an instrument that simulates the patient's functional occlusion [13].

In conclusion, acceptable baseline range of OVD for majority of individuals in any population should be 34–48 mm (3.4–4.8) cm and acceptable baseline range of MBH should be 24–37 mm (2.4–3.7) cm. In order to reconstruct the mandible and still maintain the OVD, heights of bone grafts must not be less than 2 cm or greater than 4 cm in grossly atrophic or completely lost mandible; however this can be influenced by the age and gender of the patient.

## Figures and Tables

**Table 1:** Distribution of 200 subjects based on values of occlusal vertical dimension and mandibular basal height.

Values	Male (100)		Female (100)		Total (200)	
	OVD *N* (%)	MBH *N* (%)	OVD *N* (%)	MBH *N* (%)	OVD *N* (%)	MBH *N* (%)
20–29 mm	1 (0.5)	38 (19)	2 (1)	41 (20.5)	3 (1.5)	79 (39.5)
30–39 mm	23 (11.5)	55 (27.5)	35 (17.5)	58 (29)	58 (29)	113 (56.5)
40–49 mm	72 (36)	7 (3.5)	62 (31)	1 (0.5)	134 (67)	8 (4)
50–59 mm	4 (2)	0 (0)	1 (0.5)	0 (0)	5 (2.5)	0 (0)

Total	100 (50)		100 (50)		200 (100)	200 (100)

**Table 2:** Mean and standard deviation values of OVD and MBH in relation to age and gender in 200 subjects.

Age range	*N* (%)	Male (mm)	Female (mm)	Sig.	Male	Female	Sig.
	200 (100)	OVD	OVD		MBH	MBH	
16–20 years	82 (41.0)	41.30 ± 5.76	39.29 ± 5.45	0.000	30.23 ± 6.03	29.71 ± 5.78	0.847
21–25 years	93 (46.5)	42.59 ± 5.03	40.14 ± 4.99	0.602	31.00 ± 5.87	30.16 ± 4.55	0.656
26–30 years	25 (12.5)	42.75 ± 5.14	39.40 ± 5.99	0.788	30.06 ± 7.31	26.70 ± 5.52	0.139

**Table 3:** Mean and standard deviation values of OVD AND MBH for both genders in the sample population.

	Mean ± Std. deviation	Std. error	Minimum value	Maximum value	Interquartile range
Male					
OVD (male)	42.10 ± 5.34	.53362	29.00	53.00	24.00
MBH (male)	30.54 ± 6.13	.61290	20.00	43.00	23.00
Female					
OVD (female)	39.72 ± 5.25	.52456	29.00	50.00	21.00
MBH (female)	29.63 ± 5.23	.52294	20.00	47.00	27.00

**Table 4:** Comparative values of occlusal vertical dimension in various studies.

Authors	Year	Occlusal vertical dimension	
		Male (mm)	Female (mm)
Didia and Dappa [14]	2005	69.0	63.0
Oladipupo et al. [15]	2008	71.2	65.0
Ebeye et al. [16]	2009	67.5	63.6
Oladipo et al. [17]	2014	70.2	67.4
Present study	2015	42.1	39.7
